# Localizing targets for neuromodulation in drug-resistant epilepsy using intracranial EEG and computational model

**DOI:** 10.3389/fphys.2022.1015838

**Published:** 2022-10-20

**Authors:** Yang Liu, Chunsheng Li

**Affiliations:** Department of Biomedical Engineering, School of Electrical Engineering, Shenyang University of Technology, Shenyang, China

**Keywords:** neural computational model, neuromodulation, drug-resistant epilepsy, intracranial EEG, optimal target

## Abstract

Neuromodulation has emerged as a promising technique for the treatment of epilepsy. The target for neuromodulation is critical for the effectiveness of seizure control. About 30% of patients with drug-resistant epilepsy (DRE) fail to achieve seizure freedom after surgical intervention. It is difficult to find effective brain targets for neuromodulation in these patients because brain regions are damaged during surgery. In this study, we propose a novel approach for localizing neuromodulatory targets, which uses intracranial EEG and multi-unit computational models to simulate the dynamic behavior of epileptic networks through external stimulation. First, we validate our method on a multivariate autoregressive model and compare nine different methods of constructing brain networks. Our results show that the directed transfer function with surrogate analysis achieves the best performance. Intracranial EEGs of 11 DRE patients are further analyzed. These patients all underwent surgery. In three seizure-free patients, the localized targets are concordant with the resected regions. For the eight patients without seizure-free outcome, the localized targets in three of them are outside the resected regions. Finally, we provide candidate targets for neuromodulation in these patients without seizure-free outcome based on virtual resected epileptic network. We demonstrate the ability of our approach to locate optimal targets for neuromodulation. We hope that our approach can provide a new tool for localizing patient-specific targets for neuromodulation therapy in DRE.

## 1 Introduction

Epilepsy is a neurological disease caused by disorder of the brain network (Terry et al., 2012; Lam et al., 2016). It has the characteristics of recurrent seizures, which often bring irreversible brain damage and affect the normal life of patients with epilepsy (Trinka et al., 2015). About 70% of patients can be cured by taking antiepileptic drugs, and 30% of them will develop drug-resistant epilepsy (DRE) (Kwan and Brodie, 2000; Stephen et al., 2006; Brodie et al., 2012). Patients with DRE can be treated with surgery (Choi et al., 2008) or neuromodulation (Schulze-Bonhage, 2017; Davis and Gaitanis, 2020; Sisterson and Kokkinos, 2020), such as transcranial magnetic stimulation (TMS) (Davis and Gaitanis, 2020), transcranial focused ultrasound (tFUS) (Lin et al., 2020; Zou et al., 2020). In neuromodulation therapy, different brain regions or nerves can be chosen as target, such as the vagus nerve (Stern et al., 2021), thalamus (Ryvlin and Jehi, 2021), hippocampus (Abouelleil et al., 2022), or localized epileptogenic zone (Tsuboyama et al., 2020; Rincon et al., 2021). Neurologists use intracranial EEG (iEEG), MRI and other methods combined with clinical experience to define the brain regions responsible for seizure generation and resect these regions to prevent seizure. About 30% of patients with DRE failed to achieve seizure freedom after surgical intervention (Janszky et al., 2005; de Tisi et al., 2011). Most of them are not suitable for further surgery because the suspected brain regions have been damaged. Neuromodulation is a promising technique for these non-seizure-free patients. However, to our best knowledge, the reports of localizing neuromodulatory targets for patients who failed to achieve seizure freedom are few.

EEG is widely used in the diagnosis of epilepsy. Comparing with scalp EEG, iEEG electrodes need to be embedded in the patient’s skull. The intracranial electrodes are closer to the epileptogenic zone (Kovac et al., 2017), which facilitates subsequent resection of the epileptogenic area. IEEG recording techniques include subdural grids, strips, and depth electrodes. For different epilepsy patients, different iEEG techniques need to be selected (Kovac et al., 2017).

IEEG recordings reflect the characteristics of epileptic networks and have the function of localizing epileptogenic tissues in epilepsy patients (van Diessen et al., 2013; Taylor et al., 2015; Sinha et al., 2017). Network methods can be used to extract the epileptic network, such as Pearson correlation, Granger causality (Coben and Mohammad-Rezazadeh, 2015; Sinha et al., 2017). The coefficients of Pearson correlation represent the correlation between variables. Sinha et al. (2017) calculated the coefficients between different EEG channels and used them as undirected connectivity of the epileptic model. Granger causality explores direct or indirect relationships between variables. Directed transfer function (DTF) computes interactions between input signals in frequency domain (Franaszczuk et al., 1985). A variety of network characteristics can be quantified on the extracted network matrix. One single network feature cannot fully explain all the properties of the network (van Diessen et al., 2013). Different features of network were used in epilepsy studies (Sinha et al., 2017, 2021; Paldino et al., 2019). Seizure is a dynamic process. It is difficult to explore dynamical behaviors of the original signals based on the extracted network matrix. The quantified features of epileptic networks cannot comprehensively describe the dynamics of seizure onset and termination.

Neural computational model can be used to better simulate the dynamical process of seizure (Proix et al., 2018; Saggio et al., 2020; Sip et al., 2022). Richardson proposed a method of combining dynamics and connectomics to explain the abnormal dynamics of epileptic networks (Richardson, 2012). Lopes da Silva et al. (2003) modeled transition states between normal and epileptic states for predicting epileptic pathways. Creaser et al. (2002) modeled the node dynamics and the coupling relationship between nodes, and obtained the transient dynamics during epileptic seizures. Numerous models have been used to explain the physiology of epilepsy or epileptic activity (Wendling et al., 2016). In computational models, the state of the system is commonly changed by adjusting either excitatory or inhibitory parameters, such as Z6 model (Benjamin et al., 2012) and Epileptor model (Proix et al., 2018). In the Z6 model, the values of different excitatory parameters determine whether the system is in normal or epileptic state.

In this paper, we propose a novel approach for localizing targets for neuromodulation in patients with DRE, especially for patients without achieving seizure freedom after surgery. The patient-specific epileptic network is reconstructed using multi-unit computational model. The most effective node for neuromodulation in preventing seizure is localized by introducing external stimulation. The effectiveness of our proposed approach is validated on a multi-variate autoregressive (MVAR) model. Then we validate the approach on a group of DRE patients with iEEG recordings. Finally, the candidate targets for neuromodulation are provided using the proposed approach and virtual resected network of those DRE patients.

## 2 Methods

### 2.1 Data and subject description

IEEG recordings from 11 patients were analyzed in this study. The datasets were obtained from the IEEG public website (http://www.ieeg.org), and all patients with DRE had received surgical treatment. Three patients are in seizure-free group with good outcome, who were scored as international league against epilepsy (ILAE) 1 (completely seizure-free) or 2 (no seizures, only auras) (Wieser et al., 2001). The other eight patients are in non-seizure-free group with poor outcome, who were scored as ILAE 3–6 (non-seizure-free). Interictal iEEG recordings of 10 min duration are chosen several hours away from any seizure. The iEEG data are divided into segments of 1 s duration. Each segment overlaps the previous one by 0.5 s. The sampling rate of recordings is 500 Hz. We evaluate the overlapping between the localized target nodes and the resected regions.

### 2.2 Directed transfer function with surrogate analysis

The directed transfer function (DTF) is a multi-channel directional measurement method based on Granger causality and autoregressive models (Franaszczuk et al., 1985). This method calculates the causal connection matrix between multi-channel EEG signals and measures the causal relationship between channels. The multichannel EEG process in the framework of autoregressive model (AR) can be described by the following equation (Franaszczuk et al., 1985; Kaminski and Blinowska, 1991):

$$\overset{p}{\underset{j=0}{\sum }}A_{j}x\left(t-j\right)=w\left(t\right),$$ (1)

where *x*(*t*) = [*x*_1_(*t*), *x*_2_(*t*), …, *x*_*N*_(*t*)] is the vector of EEG *N*-channel process, *p* is the order of the model. *A*_0_ is identity matrix, *A*_1_, *A*_2_, … , *A*_*p*_ are the *N* × *N* matrices of model coefficients, *w*(*t*) = [*w*_1_(*t*), *w*_2_(*t*), …, *w*_*N*_(*t*)] is the vector of multivariate zero mean uncorrelated white noise process. We use the order selection criteria of Akaike’s Final Prediction Error (FPE) criterion implemented in ARFIT toolbox (Akaike, 1971; Schneider and Neumaier, 2001).

The coefficients *A*_*j*_ can be obtained from (1) by multiplying its both sides by $x_{t-s}^{T}$ , where *x* ^*T*^ is transposed vector of *x*. We get following equation (Kaminski and Blinowska, 1991):

$$R\left(-s\right)+A_{1}R\left(1-s\right)+\cdots +A_{p}R\left(p-s\right)=0,$$ (2)

where $R\left(s\right)=E\left(x\left(t\right),x_{t-s}^{T}\right)$ is the covariance matrix with lag *s* for the vector *x*, *E* means expectation value. Applying the z-transform to the both sides of Eq. 1 (Franaszczuk et al., 1985), we have

$$X\left(z\right)=H\left(z\right)W\left(z\right).$$ (3)

where *H*(*z*) is the transfer function. Set *z* ^−1^ = *e* ^−*i*2*πf*Δ*t*^, where *f* is the frequency, Δ*t* is the sampling interval. Then we get *H*(*f*), where *H*_*ij*_(*f*) is the directed causal relationship from the node *j* to the node *i*.

The directional characteristic of the information flow from node *j* to node *i* is defined as following:

$$r_{ij}^{2}\left(f\right)=\frac{{\left|H_{ij}\left(f\right)\right|}^{2}}{\sum_{r=1}^{n}{\left|H_{ir}\left(f\right)\right|}^{2}}.$$ (4)

Note that the value of $r_{ij}^{2}\left(f\right)$ is between 0 and 1.

Surrogate data is a statistical method of analyzing nonlinear signals that facilitates the analysis of EEG signals (Dolan and Spano, 2001). We generate surrogate signals by assigning the phase of the EEG signal randomly in 200 times. The strongest 5% of the total possible causal connection are kept for further analysis. The network characteristics in high frequency gamma band are most closely correlated with improved postsurgical outcome (Wilke et al., 2011). Our preliminary study on seizure-free group also showed similar results. In this study, the network analysis focuses on gamma rhythm (31–80 Hz).

### 2.3 Other methods to build brain network

Besides DTF, there are other ways to build brain networks. The Pearson correlation coefficient (PCC) reflects the linear correlation between iEEG channels. We divide the iEEG into 1 s data segments. The PCC calculates the degree of linear correlation between two variables. It is the ratio of the covariance and standard deviation between two signals, as shown in the following:

$$P_{a,b}=\frac{\mathrm{cov}\left(a,b\right)}{\sigma_{a}\sigma_{b}},$$ (5)

where *P*_*a*,*b*_ represents the degree of linear correlation between *n* dimensional signal *a* and *b*.

Partial directed coherence (PDC) analyzes the connectivity between multi-channel signals, which is also based on Granger causality. The calculation method of *p* and *A*_*j*_ is the same as that of DTF. The transfer function ${\bar{H}}_{ij}\left(f\right)$ of PDC is calculated by the following equation (Baccala and Sameshima, 2001):

$${\bar{H}}_{ij}\left(f\right)=I-\sum_{j=1}^{p}A_{j}e^{-i2\pi f\Delta t},$$ (6)

where *I* is the identity matrix.

Isolated effective coherence (iCoh) is similar to PDC. This method computes the interrelationships of directly related nodes, but zeros out all other indirect causal relationships (Pascual-Marqui et al., 2014). When we compute the causal relationship from node *j* to node *i*, other nodes except node *j* and node *i* are called irrelevant nodes. Node *j* is the relevant nodes of node *i*.

The weighted phase lag index (wPLI) measures the phase correlation between signals by weighting the cross-spectrum of the phases of the two signals. First, we need to calculate the phase lag index (PLI) between the signals (Li et al., 2021):

$$PLI=|\mathrm{sgn}\left(\sin\left(\Delta \theta \left(t\right)\right)\right)|,$$ (7)

where sgn represents the sign function, |⋅| is absolute value, Δ*θ*(t) represents the instantaneous phase difference between the input signal *s*_1_ and the output signal *s*_2_. Then, wPLI is calculated to quantify the phase agreement between the signals (Li et al., 2021),

$$wPLI=\frac{\left|A_{1}A_{2}\sin\left(\Delta \theta \left(t\right)\right)\right|}{\left|A_{1}A_{2}\sin\left(\Delta \theta \left(t\right)\right)\right|},$$ (8)

where *A*_1_ and *A*_2_ are the corresponding amplitudes of the *s*_1_ and *s*_2_, respectively.

Relative entropy is an asymmetric measure (Kullback and Leibler, 1951), also known as Kullback-Leibler divergence (KLDIV), quantifies the difference between two signals. *x*(*t*) is divided by 1 s and overlapped by 50%, and then subjected to short-time Fourier transform to obtain *X* (*n*, *f*). The normalized spectrogram is shown in Eq. 9.

$$W_{x}\left(n,f\right)=\frac{|X\left(n,f\right){|}^{2}}{{\sum_{n,f}|X\left(n,f\right)|}^{2}},$$ (9)

Suppose *W*_*y*_(*n*, *f*) is the normalized spectrum of the signal *y*(*t*), the KLDIV from *W*_*x*_ (*n*, *f*) to *W*_*y*_(*n*, *f*) is as follows:

$$D_{KL}\left(W_{x},W_{y}\right)=\underset{n,f}{\sum }W_{x}\left(n,f\right)\log\frac{W_{x}\left(n,f\right)}{W_{y}\left(n,f\right)}.$$ (10)

KLDIV is asymmetric. When its value is higher, the difference between the signals is larger.

### 2.4 MVAR model

The electrophysiological activity in short duration can be viewed as a MVAR process. In this study, the MVAR model is written in the following differential form (Baccala and Sameshima, 2001),

$$\left\{\begin{array}{c} X_{1}\left(n\right)=0.95\sqrt{2}X_{1}\left(n-1\right)-0.9025X_{1}\left(n-2\right)+w_{1}\left(n\right),\\ X_{2}\left(n\right)=0.5X_{1}\left(n-2\right)+w_{2}\left(n\right),\\ X_{3}\left(n\right)=-0.4X_{1}\left(n-3\right)+w_{3}\left(n\right),\\ X_{4}\left(n\right)=-0.5X_{1}\left(n-2\right)+0.25\sqrt{2}X_{4}\left(n-1\right)+0.25\sqrt{2}X_{5}\left(n-1\right)+w_{4}\left(n\right),\\ X_{5}\left(n\right)=-0.25\sqrt{2}X_{4}\left(n-1\right)+0.25\sqrt{2}X_{5}\left(n-1\right)+w_{5}\left(n\right), \end{array}\right.$$ (11)

where *X*_*i*_, *i* = 1, 2, … , 5, represents the *i*th node of the network, and *w*_*i*_, *i* = 1, 2, … , 5, is the white noise. The coefficients between nodes represent the causual relationships of different nodes.

In order to find the method with best performance for constructing epileptic network and validate the effectiveness of our proposed approach, we use a five-node MVAR model to simulate the causal relationship between nodes. The time course of activity assigned on each node is generated by model (11). Simplified MVAR model could be used to simulate epileptic sources (Hosseini et al., 2018). There are both unidirectional and bidirectional connections in model (11). Node *X*_1_ is simulated as epileptic node where seizure starts. Node *X*_2_, *X*_3_ and *X*_4_ are neighbor nodes., and node *X*_4_ has bidirectional connection with node *X*_5_, which represents remote normal tissue.

There are different types of measures to construct the brain network. We choose several commonly used network measures based on causality, coherence, or information theory. We also construct the causal network combining with surrogate analysis to determine which method matches the original network best. We compare nine methods for constructing causal network, including PCC, DTF, DTF with surrogate analysis (DTF-SA), PDC, PDC with surrogate analysis (PDC-SA), iCoh, iCoh with surrogate analysis (iCoh-SA), wPLI and KLDIV. We compute the correlation coefficients between the extracted connectivity matrix and the ground truth of the model (11), which are then normalized by the maximum value. According to the value of the correlation coefficient, we choose the method with the best performance to construct brain network.

### 2.5 Multi-unit computational model

The Z6 model can simulate the dynamic process of the interaction between nodes due to information transmission during epileptic seizures, and intuitively describe the state transition of nodes. This model contains a fixed point and a limit-cycle. The noise system controls one of two factors in order to control the trajectory of the system. In the noisy system, the deterministic part at the drift coefficient can be expressed by the following single complex equation (Benjamin et al., 2012):

$$\frac{dz}{dt}=f\left(z\right)\equiv \left(a|z{|}^{4}+b|z{|}^{2}+\lambda -1+i\omega \right)z,$$ (12)

where *z* is a complex parameter, *z* = *x* + *iy*. *a* and *b* are real numbers (*a* = −1, *b* = 2), *ω* controls the oscillation frequency of the system, *λ* is the possible attractors of the system. The parameter *λ* determines the state of the system, and we choose 0 < *λ* < 1. We can consider *λ* to be the excitability parameter of the system. When *λ* approaches 1, the system is more excitable (Benjamin et al., 2012).

The nodes of the brain network have the characteristic of bidirectional functional connectivity, forming a network of interconnected nodes. We extend the equation to a network model with *N* nodes:

$$\frac{dz_{i}\left(t\right)}{dt}=f\left(z_{i}\right)+\beta \overset{N}{\underset{j\neq i}{\sum }}KG_{ij}\left(z_{j}-z_{i}\right)+\alpha w_{i}\left(t\right),$$ (13)

where *G*_*ij*_ is the normalized information connectivity matrix between nodes, *w*(*t*) represents white noise with a mean of 0.0003 and a standard deviation of 0.05 (Sinha et al., 2017), *α* is the coefficient of noise. *β* equals 0.02 here. In order to achieve the same order of magnitude as the undirected symmetric information-connected matrix (Benjamin et al., 2012), the normalized matrix *G* is then multiplied by *K* = 1,000. The connectivity matrix *G* describes the topology of the epileptic network, and determines the interaction between each node of the system. When iEEG dataset is analyzed, the patient’s epileptic networks are constructed by network measures, which is then used as the matrix G of the computational model. In this case, the dynamic behavior of the model is determined by patient’s specific brain network. The model was solved numerically using a fixed step Euler-Maruyama solver with a step size of 0.05.

During the numeric simulation, the network is driven by random noise. The time for a node to change from a stationary state to an oscillating state is called the escape time (*T*_*es*_) (Sinha et al., 2017). *T*_*es*_ is used as an indicator for predicting seizure (Benjamin et al., 2012). We use the Z6 model to simulate the epileptic brain as a bi-stability state network (Goodfellow and Glendinning, 2013; Sinha et al., 2017). The probability of a node entering epileptic state is inversely proportional to *T*_*es*_ (Petkov et al., 2014; Sinha et al., 2017). It is also proportional to the stability of the system, and the value of *T*_*es*_ decreases as the parameter *λ* increases (Benjamin et al., 2012). The parameters *λ* of all nodes are set to *λ*_0_ or *λ*_1_ on non-target or target nodes, respectively. The optimal *λ*_0_ and *λ*_1_ are chosen by grid search of *λ*_0_ − *λ*_1_ pairs. *λ*_0_ is chosen between 0 and 0.5 in step of 0.05, and *λ*_1_ is chosen between 0.5 and 1 in step of 0.05. The Δ*T* is calculated at each *λ*_0_ − *λ*_1_ pair. The optimal *λ*_0_ and *λ*_1_ are found when there is the largest standard deviation of Δ*T* for all pairs. First, we set the *λ* of all nodes to *λ*_0_, and record the *T*_*es*_ as *T*_0_. Then, we change the *λ* of each target node to *λ*_1_, and record the *T*_*es*_ as *T*_1_. The difference between *T*_0_ and *T*_1_ is the change in escape time (Δ*T*), $\Delta T=\left|T_{0}-T_{1}\right|$ , represents the effectiveness of the neuromodulation applied on a given node.

### 2.6 Localizing targets for neuromodulation

**FIGURE 1:**
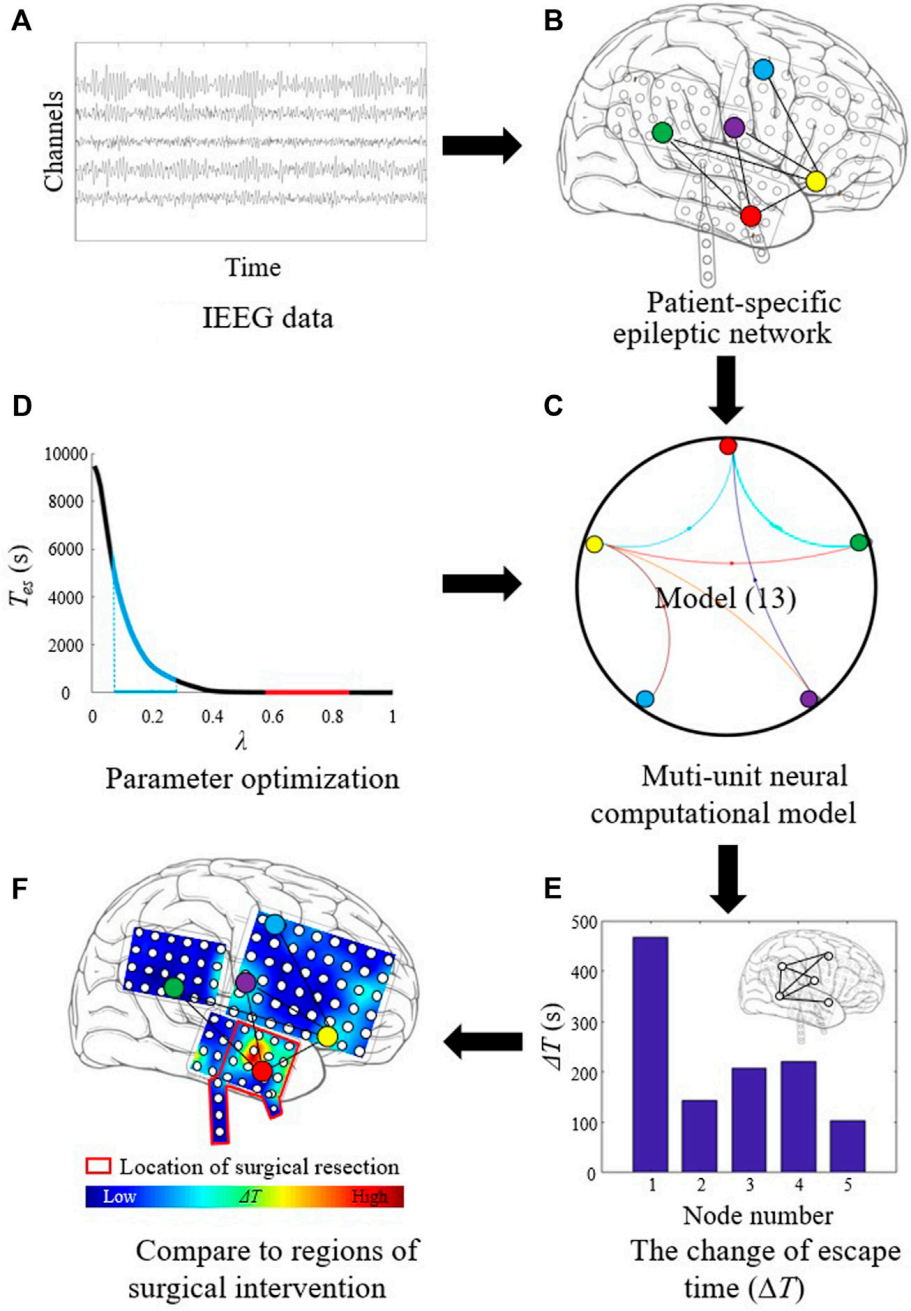
Procedure of localizing target for neuromodulation in drug-resistant epilepsy. **(A)** The segmented iEEG recordings. **(B)** Patient-specific epileptic network based on iEEG data. **(C)** Multi-unit neural computational model based on the epileptic network and the Z6 model. **(D)** Parameter optimization for *λ*. **(E)** Calculating the change of escape time (Δ*T*) of each node. **(F)** Localizing optimal target with the largest Δ*T* value.

The procedure of localizing targets for neuromodulation is shown in Figure 1. First, the segmented iEEG recordings are used, as shown in Figure 1A. The data are processed to construct patient-specific epileptic network (Figure 1B). The epileptic network is in the form of causal connectivity matrix. The multi-unit neural computational model is constructed based on the epileptic network and the Z6 model (Figure 1C). The number of nodes of the multi-unit model is same as the number of channels in iEEG the recordings. The optimal values of *λ* are determined (Figure 1D). Using the selected parameters, the Δ*T* of each node is then calculated (Figure 1E). The distribution of Δ*T* is plotted on patient’s head model, and the node with the largest value of Δ*T* is selected as optimal target. The optimal target is compared with the resected regions of epilepsy patient, as shown in Figure 1F.

### 2.7 Validation of neuromodulation

We calculated *T*_*es*_ of epileptic brain networks in all patients with inhibitory modulation on the localized target nodes and non-target nodes. The Wilcoxon rank sum test was used (Wilcoxon, 1945), and *p* < 0.01 was chosen as significance threshold. The proposed approach was then performed to localize candidate targets for neuromodulation in patients without seizure-free outcome.

## 3 Result

### 3.1 Localizing the critical node of MVAR model

**FIGURE 2:**
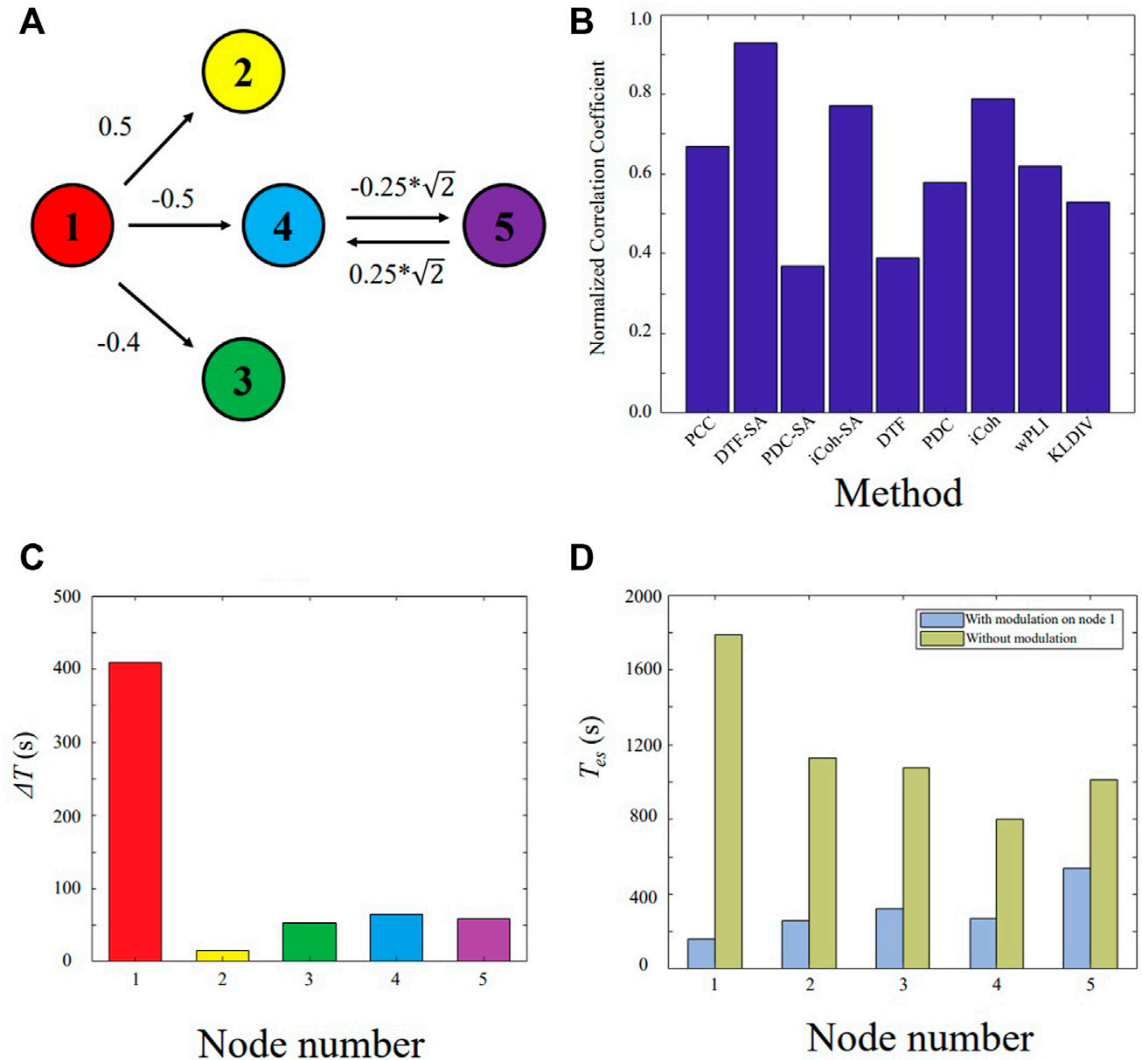
Validating the proposed approach on the five-node model. **(A)** Five-node causal network. **(B)** Normalized correlation coefficient between the constructed network and the ground truth. **(C)** Δ*T* for each node. **(D)** The *T*_*es*_ value of the network with and without external modulation.

The model (11) is shown in Figure 2A. The node *X*_1_ is the main driven force for the model to enter oscillatory state, which is simulated as the epileptogenic node. The normalized correlation coefficients of nine different methods are plotted in Figure 2B. Based on the extracted causal connectivity by DTF-SA method, we localize the target for neuromodulation using our proposed approach. The optimal values of *λ*_1_ and *λ*_0_ is 0.85 and 0.50, respectively. The result of Δ*T* is shown in Figure 2C, and the node *X*_1_ is with the highest value. The effectiveness of external modulation on the node *X*_1_ is shown in Figure 2D. The *T*_*es*_ of the network decreases significantly while the external excitatory stimuli is applied on node *X*_1_.

### 3.2 Localizing targets for neuromodulation using patient data

The localized targets for neuromodulation of 11 patients are listed in Table 1. For patient P1-P3 with seizure-free outcome, the targets are inside the resected regions. In non-seizure-free group, localized targets for patients P4, P10, and P11 are outside the resected regions, and localized targets for patients P5-P9 are inside the surgical resected regions. The mean error distance is 8.4 mm in non-seizure-free group. The values of parameter *λ*_0_ for 11 patients is 0.22 ± 0.08, *λ*_1_ is 0.99 ± 0.01 (mean ± SD).

**TABLE 1:** Patient information, surgical results, optimal parameters, and target location.

Patient	Dataset ID	Surgical outcome	Target before resection	Parameter *λ*_0_	Distance to resection (mm)	Target after resection	Distance to resection (mm)
P1	Study 038	Seizure free	ITS2	0.25	0.0	-	-
P2	Study 021	Seizure free	RTG15	0.30	0.0	-	-
P3	Study 026	Seizure free	LFG48	0.30	0.0	-	-
P4	Study 028	Not seizure free	LPG7	0.30	28.0	LPG7	28.0
P5	Study 004–2	Not seizure free	RAT4	0.15	0.0	RG20	14.1
P6	Study 016	Not seizure free	RTG24	0.20	0.0	RFG19	45.0
P7	Study 029	Not seizure free	AIT4	0.20	0.0	LT12	30.0
P8	Study 020	Not seizure free	RAG20	0.15	0.0	RAG6	10.0
P9	Study 019	Not seizure free	LT9	0.25	0.0	LF10	48.7
P10	Study 022	Not seizure free	TSG7	0.20	20.0	TSG7	20.0
P11	Study 033	Not seizure free	LTG7	0.35	19.3	LTG7	19.3

**FIGURE 3:**
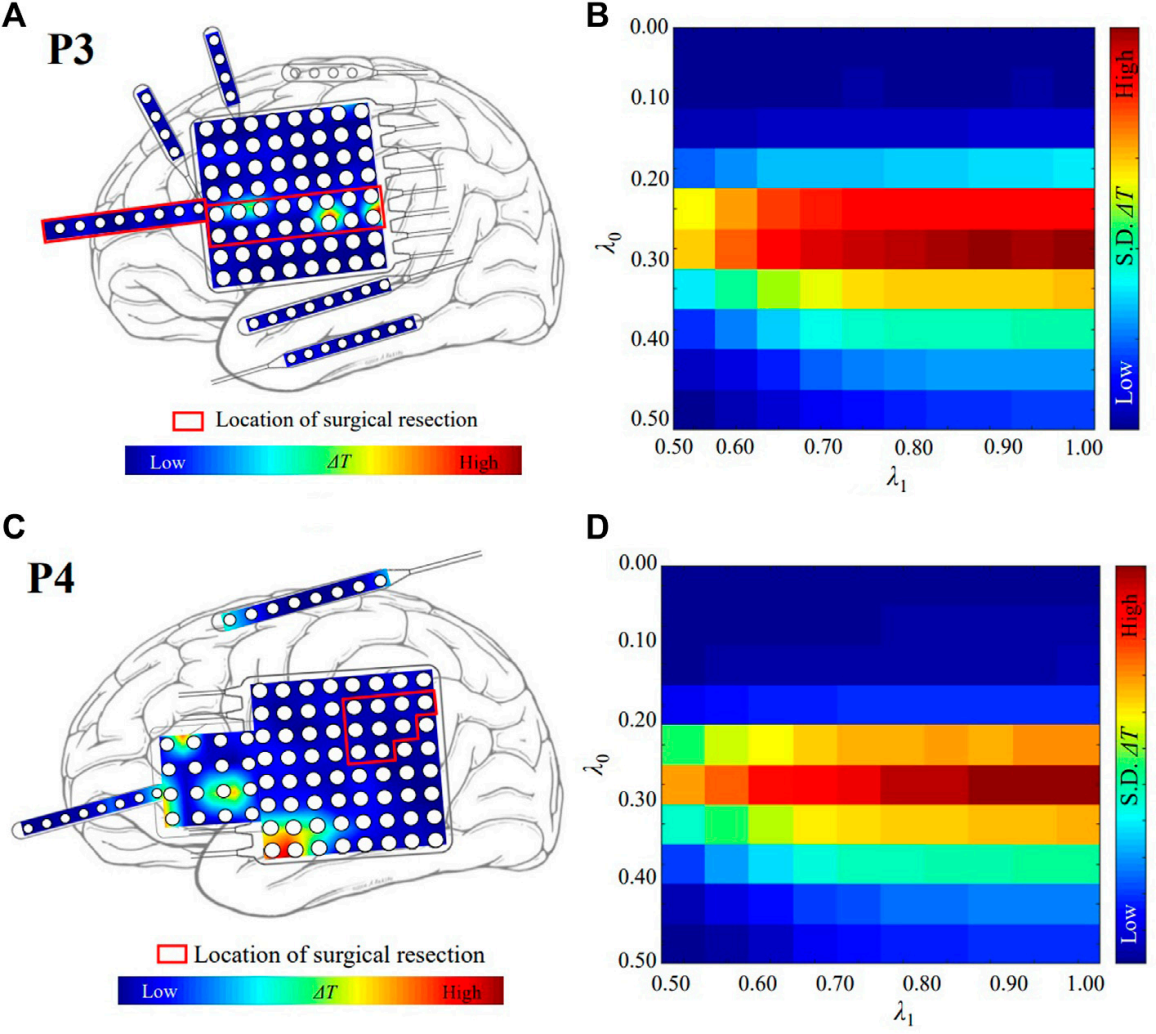
The localized targets for neuromodulation of patient P3 and P4. **(A)** The distribution of Δ*T* for patient P3. The white circles represent the contacts of the electrodes. Red and blue indicate the magnitude of the value of Δ*T*. The red color indicates that the value of Δ*T* is large. The blue color indicates that the value of Δ*T* is small. **(B)** The optimal value of *λ* for patient P3. *λ*_0_ = 0.30 and *λ*_1_ = 1.00. The red color indicates that the value of standard deviation (S. D.) of Δ*T* is large. **(C)** The distribution of Δ*T* for patient P4. **(D)** The optimal value of *λ* for patient P4. *λ*_0_ = 0.30 and *λ*_1_ = 1.00.

The localized targets for neuromodulation of patient P3 and P4 are plotted in Figures 3A,C, respectively. Red region indicates the stimulation is effective to suppress seizure in Figures 3A,C. Patient P3 belongs to the seizure-free group. The resected region of this patient was mainly in left lateral frontal cortex. The electrode LFG48 with the highest Δ*T* is selected as the target, and reside in the resected region (red rectangle), as shown in Figure 3A. Patient P4 belongs to the non-seizure-free group. The resected region was mainly in left parietal cortex. The electrode LPG7 with the highest Δ*T* is selected as the target, which is 28.0 mm away from the resected region (red rectangle), as shown in Figure 3C. The distribution of Δ*T* for choosing optimal *λ*_0_−*λ*_1_ pair is plotted in Figures 3B,D. The *λ*_0_ = 0.30 and *λ*_1_ = 1.00 for both patients.

**FIGURE 4:**
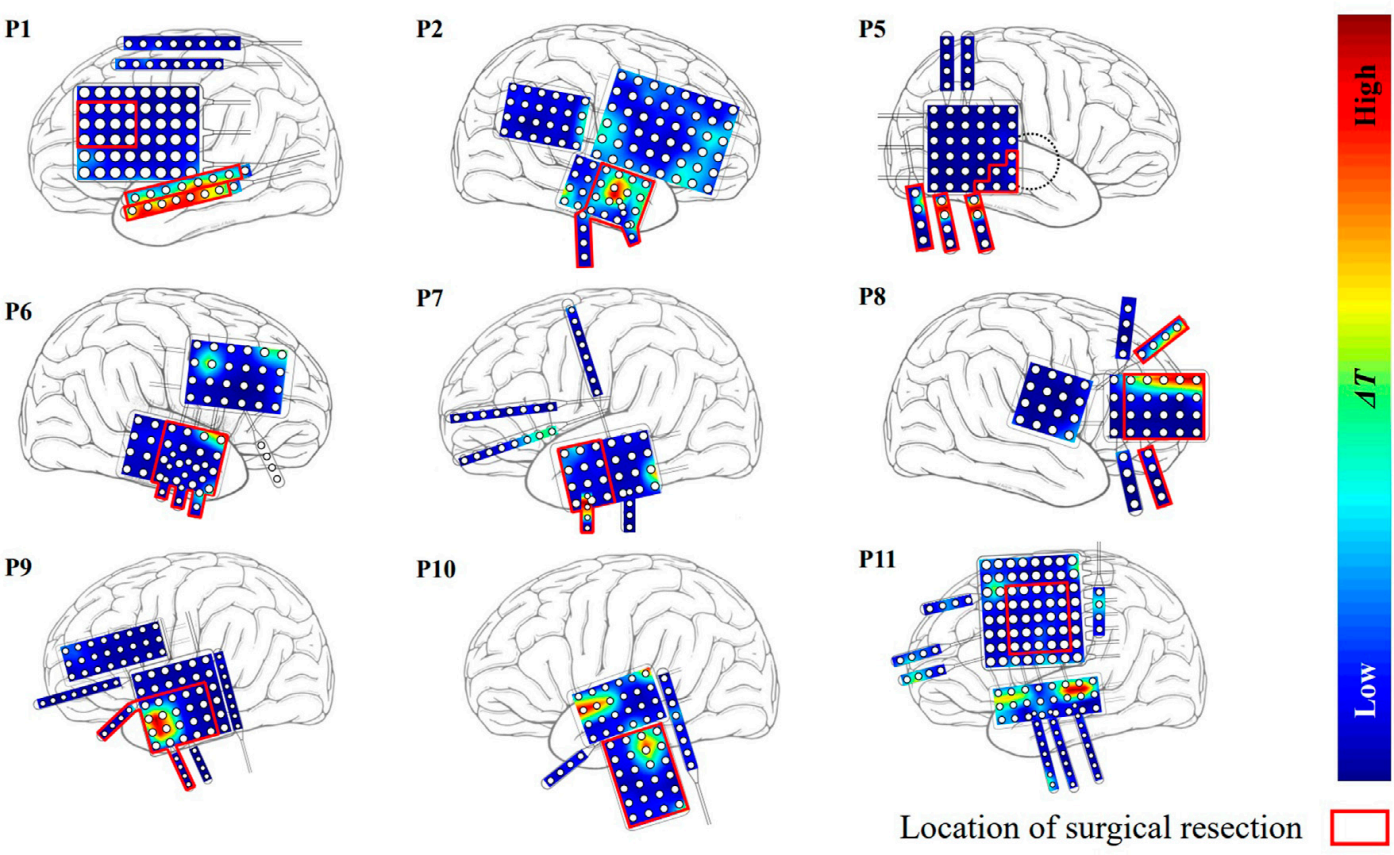
The distribution of Δ*T* for the other nine patients except P3 and P4. Red region indicates that the effect of modulation is strong.

The Δ*T* distributions for the other 9 patients are plotted in Figure 4. Among them, patients P1 and P2 belonged to the seizure-free group. The surgical field in both patients was in the left temporal lobe. Targets identified by our method that were effective in eliminating epilepsy were located at the surgical field of each of the two patients.

### 3.3 The effectiveness of neuromodulation

**FIGURE 5:**
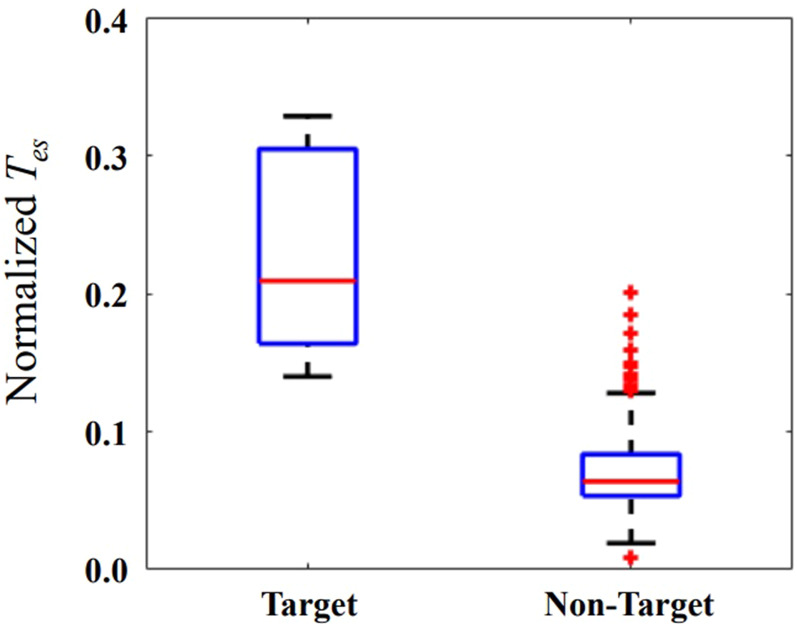
The normalized *T*_*es*_ when external modulation is applied on the localized target and non-target nodes. The horizontal red bar represents the median, and the blue box represents the rang from first to third quartile, respectively. The horizontal black lines represent the upper and lower limits, and the red plus sign represents outlier data.

The *T*_*es*_ is normalized relative to its minimum and maximum values of each patient. The horizontal red bar represents the median in Figure 5. The median of the *T*_*es*_ is 0.21 and 0.06 for target and non-target nodes, respectively. The values of *T*_*es*_ on the localized target nodes are significantly longer than the values on non-target nodes (*p* < 0.01). The modulation on the localized target nodes is more effective in suppressing seizure than non-target ones. Outlier data indicate that modulation on some non-target nodes is more (or less) efficient than modulation on other nodes.

We remove the iEEG channels in the resected regions, and reconstruct virtual resected networks for the non-seizure-free patients. In the virtual resected network, the iEEG channels in resected regions are removed. The connectivity matrix G is calculated using other channels, which results in a smaller matrix. The value of *λ*_0_ is set to 0.22 for the non-target of the resected network, and *λ*_1_ = 1. The localized candidate targets for neuromodulation are listed in Table 1. The mean distance between the new targets and the surgical resected regions is 26.9 mm. The localized targets for Patient P4, P10, and P11 are not changed before and after surgical resection. The mean distance between the new localized targets and the resected regions is 29.6 mm for patient P5-P9.

## 4 Discussion

Localizing the effective targets is the key to neuromodulation therapy. The proposed approach identified the node *X*_1_ of the MVAR model as optimal target successfully, which is the designed node to drive the model to oscillatory state. The network measures based on correlation, causal effects, phase lag, and information entropy were compared for reconstructing the network. The DTF-SA method showed highest similarity between the reconstructed network and the ground true. Our results could help other study for choosing network measures. We choose the Z6 model as the network node because of the relative low computational cost. Other neural computation models could also be adopted, such as Epileptor model (Proix et al., 2018). The parameter *λ*_0_ of non-target node is the only parameter need to be determined for the network except for connectivity matrix. Our result show that the mean value of *λ*_0_ for non-target nodes is good in most cases when analyzing the iEEG dataset, and the value *λ*_1_ for target node is set to 1, which leads that our proposed approach is easy to use.

Patient P1, P2, and P3 have undergone surgical resection, and achieved good outcome. The epileptogenic tissues are assumed inside the resected regions. Based on the patient-specific epileptic network, all localized targets for those patients reside in the resected regions. Those results indicate that our approach find the target responsible for seizure generation. The localized targets for patient P5-P9 also reside in the resected regions, which is consistent with judgement of neurologist. The epilepsy is a brain network disease. Resection of brain tissue changes the topology of epileptic network, and seizure may start from other brain location. Applying the external stimulation on a given node will result in influence on whole network. In this context, we believe the neuromodulation measure may lead to better outcomes for those patients without seizure-free outcome. The localized targets for patient P4, P10, and P11 are outside the resected regions based on the epileptic network before surgery, and the mean distance from the surgical resected regions is 22.4 mm. After removing the resected nodes, our approach also localizes the targets on the same electrodes. This result reflects that our proposed network is stable on localizing the targets even with the virtual resection.

When applying inhibitory stimulation on the localized target nodes, it can significantly delay the brain network from entering a state of oscillation relative to the non-target nodes, as shown in Figure 5. This demonstrates the effectiveness of the proposed method for preventing epileptic seizures. Neuromodulation is used as a non-destructive means of brain network regulation, such as TMS, tFUS, which have different spatial resolutions (Davis and Gaitanis, 2020; Lin et al., 2020; Zou et al., 2020). Considering the applicability of our proposed method, the number of targets selected for neuromodulation is 1. Selecting 2 or more targets for neuromodulation will have a more obvious modulation effect, but it is not suitable for neuromodulation methods with low spatial resolution, such as TMS.

Our method induces resting-state brain networks into epilepsy *via* stimulation parameters. This approach differs from current neuromodulation treatments. For example, tFUS suppresses seizure by reducing the excitability of the nervous system (Folloni et al., 2019; Lin et al., 2020; Zou et al., 2020). However, in clinical surgery, the traditional method is to find the epilepsy surgery area by evoking electrical stimulation. Combined with the clinical surgical process, we select parameters that can induce the brain network to enter the epileptic state to determine the target of neuromodulation. This choice ensures the practicality of our method.

Furthermore, this approach has limitations. The iEEG recording is an invasive measure mainly for presurgical evaluation of DRE patient. We have not applied this measure on scalp EEG recording. The low coverage of the intracranial electrodes on the epileptogenic zone may result in the inaccurate of constructing epileptic network, which ultimately leads to poor localization of the targets. Validating our method in the real application will further advance the technology. On the one hand, the Z6 model is a noise-driven computational model that simulates epileptic seizures (Sinha et al., 2017). Due to the randomness and uncertainty of noise, multiple calculations are required to avoid accidental factors. The long computational time is another limitation of our method. On the other hand, we chose the Z6 model to simulate the dynamic process of epileptic seizures. Other neural computational models can also replace the Z6 model, such as the Epileptor model. Therefore it is necessary to choose different and more suitable parameters to simulate the process of neuromodulation.

## 5 Conclusion

The effective neuromodulation therapy is very important for DRE patients with bad surgical outcome. The DTF with surrogate analysis is more suitable for constructing patient’s epileptic network using iEEG recording. Multi-unit computational model can be used to simulate the seizure dynamics, and evaluate the effects of external excitatory and inhibitory stimulation. By using iEEG and computational model, our study provided a new approach to localize the optimal targets for the potential neuromodulation of these patients.

## Data Availability

Publicly available datasets were analyzed in this study. This data can be found here: http://www.ieeg.org.
